# Exploring the Contextual Factors of Religious Leader Participation in Health Communication: Evidence from a Qualitative Study in Sierra Leone

**DOI:** 10.1007/s10943-022-01632-3

**Published:** 2022-08-20

**Authors:** Hanna Luetke Lanfer, Constanze Rossmann, Sorie Ibrahim Kargbo

**Affiliations:** 1 School of Public Health, Bielefeld University, Universitätsstraße 25, 33615 Bielefeld, Germany; 2 Division of Communication and Media Research, LMU Munich, Oettingenstr. 67, 80538 Munich, Germany; 3 Freetown, Sierra Leone

**Keywords:** Religious leaders, Sierra Leone, Qualitative study, Ebola outbreak

## Abstract

As there are many and sometimes ambivalent intersections of health and religion, strategic collaborations with religious opinion leaders in health campaigns have been increasingly explored. Despite the known influence of distinct contextual factors within emergency and non-emergency settings, existing research seldom distinguishes between those different factors and their impact on the inclusion of religious leaders as health messengers. To compare the contextual factors of religious leaders as health messengers during emergency and non-emergency situations in a setting with high religious affiliations, this study used a qualitative approach and triangulated the perspectives of three different samples, including (religious) opinion leaders, members of religious communities, and developers of health communication strategies in Sierra Leone. The results provide multifaceted insights into contextual factors applicable to emergency and non-emergency settings as well as the risks and opportunities. Recommendations for the incorporation of religious leaders in health promotion activities in consideration of different contextual factors are provided.

## Introduction

There is abundant evidence of the multiple intersections between religion and health; spirituality and religion are strongly aligned with people’s deepest motives and search for meaning in life (Hill & Pargament, 2003). As stewardship of one’s health is a core belief and sacred responsibility in several religions (Padela et al., 2018), behaviors that align with these beliefs can be strongly motivated (Oman, 2018). Thus, it should not come as a surprise that studies have shown a positive relationship between certain health-protective behaviors and better health outcomes in religious versus non-religious groups; for example lower tobacco use (Brown et al., 2014) and substance abuse (Hai et al., 2019). However, religious beliefs and practices are not uniformly equivalent to having a favorable impact on health.

Given the many and sometimes ambivalent intersections of health and religion, a growing number of scholars and practitioners are exploring strategic collaborations with faith-based organizations (FBOs) in health campaigns to influence health-related behaviors of religious groups and promote better health (Downs et al., 2017; Ruijs et al., 2013). For their established role in religious communities, partnerships with religious opinion leaders have become a focal point in research and practice (Adedini et al., 2018; Isiko, 2020). In this, religious leaders have served as health messengers during acute public health threats as well as non-emergency settings (Schoenberg & Swanson, 2017). While it is known from studies in the field of health communication that contextual factors differ within emergency and non-emergency situations (Reynolds & Seeger, 2005), existing research seldom distinguishes between those different contextual factors and their impact on the inclusion of religious opinion leaders as health messengers. Therefore, this study seeks to contribute to this line of research by identifying the contextual factors of emergency and non-emergency settings and the resulting challenges and opportunities for the inclusion of religious leaders as health messengers. Following the literature review and research questions, the study’s methodological approach with three different samples is introduced. Results are presented chronologically in line with the two research questions and followed by a discussion and recommendations for the incorporation of religious leaders in health promotion activities in consideration of different contextual factors.

## Literature Review

### The Role of Religious Leaders in Public Health Campaigns

Relying on non-medical laypeople such as religious leaders as multipliers in public health campaigns is not new: Since the invention of the theory of opinion leadership, or the two-step flow model (Chowdry & Newcomb, 1952; Lazarsfeld et al., 1948), opinion leaders—individuals from whom others seek advice and whose views and behaviors influence those of others—have come to play a central part in health communication research (Valente & Pumpuang, 2007). One of the most common methods of identifying opinion leaders is to do so based on their preexisting leadership roles (e.g., a pastor, imam, or Buddhist monk), as it is cost-effective and can be easily replicated in varying settings.Preexisting leadership positions are also ‘a generally agreed-on societal value’ (Valente & Pumpuang, 2007, p. 886) and thus traditionally granted more power and authority than unofficial leaders. In the case of religious leaders, authors have pointed out that independent of their religion, religious authorities are not only institutionalized in their own organizational structures but also in wider social circles and are therefore valuable partners to disseminate and promote health messages (Krishnakumar et al., 2015).

For their well-established social roles and because of the intersections of religion and health, religious leaders have been employed as opinion leaders in health campaigns for acute public health emergencies (e.g., during the COVID-19 pandemic Isiko, 2020; Wijesinghe et al., 2022) or West African Ebola outbreak (Lyons et al., 2021)) and in non-emergency settings [e.g., HIV prevention in Tanzania (Downs et al., 2017), contraceptive use in Nigeria (Adedini et al., 2018), and immunization efforts in various countries (Jalloh et al., 2020)].

### Contextual Factors of Emergency and Non-emergency Settings

Emergency and non-emergency situations can be distinguished as an immediate threat for the general public in a specific location versus a potential, modifiable risk for specific subpopulations) (Reynolds & Seeger, 2005). It is known from studies in the field of health communication that contextual factors—a ‘set of circumstances or unique factors that surround a particular implementation effort’ (Damschroder, 2009, p. 3)—differ between emergency and non-emergency situations. These differences in contextual factors may result in varying challenges and opportunities for the inclusion of opinion leaders in public health campaigns.

While various frameworks for health communication strategies refer to contextual factors in general, two recent reviews (Nilsen & Bernhardsson, 2019; Rogers et al., 2020) lament a lack of consensus and detailed conceptualization of what context is and suggest various overlapping dimensions of contextual factors. Relevant contextual factors include organizational structures, leadership, the wider environment, financial resources, policy, and patients/public (Nilsen & Bernhardsson, 2019). Against the lack of coherence over what contextual factors are, it does not come as a surprise that the contextual factors of emergency and non-emergency settings have seldom been accounted for in existing studies. This becomes even more evident with regard to the role of contextual factors in public health campaigns with religious opinion leaders as health messengers. Noticeably, previously published studies (e.g., Downs et al., 2017; Jalloh et al., 2020; Lyons et al., 2021) have largely framed the positive outcomes of engaging faith-based opinion leaders as health messengers. However, there has been little disclosure regarding the contextual factors into which they were integrated and how they may have contributed to the success of these strategies. A comparison of the contextual features in emergency and non-emergency settings and resulting opportunities and challenges for health promotion activities have received little attention. For the known relationship between religious beliefs and practices and health (both favorable and not) and the risk of unintended, possibly negative responses from audiences, the contextual factors in relation to challenges and opportunities of including religious leaders as opinion leaders in emergency and non-emergency settings require further scholarly investigation. We thus ask:

- RQ1: What are the contextual factors in a public health emergency, and the resulting challenges and opportunities for the inclusion of religious opinion leaders as health messengers?
- RQ2: What are the contextual factors in a non-emergency setting, and the resulting challenges and opportunities for the inclusion of religious opinion leaders as health messengers?

## Methods

### Study Design and Setting

This research is part of a larger study to increase health promotion for underserved communities in Sierra Leone (Luetke Lanfer, 2021; Luetke Lanfer & Reifegerste, 2021). In this qualitative study, we applied a theory-based approach, where a previously defined research question guided data collection and analysis. To increase the validity of this study and gain differentiated insights into the perspectives of distinct social and professional groups regarding the contextual factors and related opportunities and challenges for the inclusion of religious leaders in different settings, we used a triangulation approach and combined the data from three different samples (interviews with (1) developers of health communication strategies who have worked with religious leaders (*n* = 11), (2) local opinion leaders, including religious leaders (*n* = 11), and (3) eight focus group discussions with members of different religious communities (*n* = 60)).

Fieldwork for this study was conducted in November/December 2018 in Sierra Leone. The research team for this study consisted of the first author (interviews) and two trained local assistants, including the third author (focus group discussions). Focus groups were held in the local language (Krio) and interviews in either Krio or English.

### Sampling and Procedure

Data collection was carried out in Sierra Leone as the country has a long history of including religious leaders in finding solutions to health-related problems. An Interreligious Council (IRC) with Islamic and Christian leaders, representatives of the two dominant religions, was established toward the end of the country’s civil war to support peacebuilding and dealing with trauma (Conteh, 2008). As religious and other local opinion leaders were involved in the emergency of Sierra Leone’s Ebola outbreak which later led to policy changes (e.g., National Health Promotion Strategy; Kinsman et al., 2017; Marshall, 2017), this study compared the epidemic and post-epidemic phase of this public health emergency. Purposeful and snowball sampling led to the recruitment of participants for the three samples of this study.

#### Developers of Health Communication Strategies

Developers were recruited using the first author’s network of health professionals and organizations who had worked with religious opinion leaders in the area of health. This resulted in the recruitment of 11 developers with different professional backgrounds from non-governmental organizations (NGOs) (*n* = 9) and government institutions (*n* = 2). Two interviews were held via video call, the remaining face-to-face at the participants’ workplaces, mainly in the capital Freetown. They were audio-recorded and lasted between 41 and 80 min (*M* = 50.20 min). Interviewees did not receive an incentive.

#### Opinion Leaders

The health promotion strategy by the government of Sierra Leone recognizes different groups of local opinion leaders, including religious and local leaders, media workers/journalists, and medical workers who often share the same social space with religious leaders or even work with them. Including the perspectives of all these groups allows us to take into account the self and external perceptions regarding the role of religious leaders as health messengers. The IRC was contacted to recruit two high-ranking religious leaders (one bishop, one sheikh). Each interviewee was asked for a referral of a lower-ranking, community-based pastor and imam, resulting in *n* = 4 religious leaders. Other opinion leaders were recruited upon referrals by the developers. One participant was joined by a colleague, leading to a total of 11 participants in 10 interviews. Interviews were held face-to-face at a location convenient for the participant in three districts of Sierra Leone. They were audio-recorded and lasted between 20 and 45 min (*M* = 37.35 min). No incentive was given.

#### Local Members of Religious Communities

To recruit members of religious communities, contact persons in eight different locations across Sierra Leone were identified. Working with two local research assistants, the research team traveled to the locations. After asking for permission from the local chief, the contact person was asked to suggest six to eight participants based on a list of criteria (i.e., gender, different age groups, and active membership in a religious congregation). After receiving informed consent from each participant, eight same-sex focus group discussions with a total of 60 participants took place at a communal meeting point or one of the participants’ houses. All focus group discussions were carried out in Krio, audio-recorded, and lasted between 45 and 75 min (*M* = 52.25). Drinks and snacks were provided after the discussions.

### Interview and Discussion Guides

Separate semi-structured interview and discussion guides were developed to ensure that all relevant themes were discussed. Developers and opinion leaders were asked about their professional background, their experiences in strategic health communication since the country’s Ebola outbreak, and the risks and strengths of including religious leaders as opinion leaders. Focus groups with local members of religious communities revolved around the following themes: perception of health communication activities during and after the Ebola outbreak; experiences with religious leaders in the area of health; and preferred channels and messengers of health communication.

### Data Analysis

All interviews and focus group discussions were transcribed verbatim and fully anonymized. When data were collected in Krio, the translation to English and transcription process occurred simultaneously. Data were analyzed using qualitative content analysis (Mayring, 2000) and supported by the software MAXQDA. Qualitative content analysis is both concept- and data-driven and allows for data reduction into categories with similar meanings (Schreier, 2014). All three samples were analyzed separately following the same procedures: Interviews were coded by the first author in a data-driven way, new subcategories were created, reviewed, linked, aggregated, and defined to ensure that they were mutually exclusive. During selective coding, the material was reviewed and recoded based on the final coding frame. In the last step, the final coding frames of the three samples were compared. Coded transcripts were read again to determine that subcategories were suited to be matched across the three samples. Identified themes were aligned with the context dimensions ‘organizational structures, leadership, the wider environment, financial resources, policy, and patients/public’ as suggested by a recent review (Nilsen & Bernhardsson, 2019). Different stages of refinement of the coding frames were thoroughly discussed with the research assistants and co-authors.

For reporting results, participants are referred to by their sample, their gender (F for female and M for male), and a number, e.g., Developer, M1. Among opinion leaders, an additional distinction is made between ‘Religious opinion leader’ and ‘Opinion leader’ (Tables 1, 2, 3).

### Ethics, Consent, and Permission

This study was approved by the Sierra Leone Ethics and Scientific Review Committee (SLESRC22082018) and the ethical commission of Erfurt University (UE01072018) before data collection. All participants signed, or in the case of illiteracy, thumb-printed in the presence of an impartial witness, an informed consent form. All our procedures have been performed in accordance with the ethical standards as laid down in the 1964 Declaration of Helsinki and its later amendments.

## Results

This section integrates the data from all three samples and is divided into two parts. It builds on contextual factors described by Nilsen and Bernhardsson (2019) and resulting challenges and opportunities of religious leaders during a) emergency and b) non-emergency settings. A comparison of the subcategories for each identified contextual factor during emergency and non-emergency settings is displayed in Table 4.

**Table 1:** Sectors and professional positions of developers (*n* = 11)

Code	Sector	Professional position	Gender
DM1	NGO	Health program manager	Male
DM2	NGO	Health program manager	Male
DM3	NGO	Health program manager	Male
DM4	NGO	Health program coordinator	Male
DM5	NGO	Health program coordinator	Female
DF6	NGO	Manager of media health programs	Male
DM7	NGO	Manager of media health programs	Male
DM8.1 &8.2	NGO	Health program coordinators	Male
DM9	Government	Health education division, national level	Male
DM10	Government	Health education division, district level	Male

**Table 2:** Professional sectors of opinion leaders (*n* = 11)

Code	Sector	Gender
ROLM1	Bishop	Male
ROLM2	Sheikh	Male
ROLM3	Pastor	Male
ROLM4	Imam	Male
OLM5	Journalist urban	Male
OLM6	Journalist rural	Male
OLM7	Medical worker urban	Male
OLF8.1 & 8.2	Medical workers rural	Females
OLM9	Local leader urban	Male
OLM10	Local leader rural	Male

**Table 3:** Demographics of members of religious communities (*n* = 60)

Characteristics	*n*
*Gender*	
Female	30
Male	30
*Location*	
Rural	32
Urban	28
*Age group*	
18–30	24
31–45	20
46+	11
Missing	5
*Education*	
No formal education	31
Primary school	15
Secondary school	13
College/University	1
*Religious affiliation*	
Muslim	41
Christian	19

**Table 4:** Comparison of context factors during emergency and non-emergency setting

Context factors^a^	Emergency setting	Non-emergency setting
Organizational structures	Existing, dense network of religious bodies	Ambivalent reputation as reliable messengers for health information
Leadership	Public statements of religious scholars on conflicting issues	Conflicting topics and priorities
Wider environment	Focus on one health topic	Multitude of health topics with complex messages
Financial resources	Available funding	Lack of funding
Policy	Integration into well-functioning response mechanisms	Few legal regulations and inconsistent services
Patients/public	Need of hope in times of despair	Ambiguity of accepting fate versus taking action

^a^Context factors are based on ‘description of context dimensions’ by Nilsen and Bernhardsson (2019)

### RQ1: Emergency Setting (West African Ebola Outbreak)

Results indicate that the contextual features of the Ebola epidemic largely supported the inclusion of religious leaders as health messengers: Due to an existing dense network of religious bodies throughout the country (organizational structures) and public statements of religious scholars on conflicting issues (leadership) in times of despair (patients/public), religious leaders were believed to have increased message exposure in the local population and people’s willingness to comply with the new emergency regulations. Simultaneously, the messages of religious leaders were strengthened by other characteristics of the emergency response, such as a focus on one health topic (wider environment), available funding (financial resources), and an integration into well-functioning response mechanisms (policy), which allowed for trainings, supervision and financial incentives as well as supporting legal force.

#### Existing Dense Network of Religious Bodies

Once religious leaders alongside other opinion leaders were recognized as influential and trusted sources of information for the public, they were easy to identify due to an existing, dense structure of religious institutions that would reach even remote audiences:

You may go to a village where there is no school or there is no electricity. But you can hardly see a village where there is no mosque or a church. (Developer, M2)

While there was an awareness that technical and medical interventions all contributed to a decline in infections, participants claimed that the inclusion of key opinion leaders had been key to changing the trajectory of the epidemic:

When our own people started telling us about this Ebola, it got better because everybody could understand. (Religious member, M5).

#### Public Statements of Religious Scholars on Conflicting Issues

The touching and washing of corpses during burial traditions became known as a primary source of infection with the Ebola virus and were thus prohibited. As this prohibition contradicted traditional and religious rituals, high-ranking Islamic and Christian scholars were asked to study their religious sources and offer supporting statements for the new law:

We told them they had to go to the Bible and Qur’an again and they found out that, for example, the Qur’an says during the time of the holy wars, you know, the prophet himself, he buried dead bodies, even the disciples, without washing them because that was not a normal situation. And that was what they used as the entry point: Ebola time was not a normal time …. If by washing the dead bodies you are putting your life at risk, then you should not because the Bible and the Qur’an also say that you should not unnecessarily put your life at risk or the life of other people. So that´s how they go, they identify all those verses and scriptures to support the public health messages. (Developer, M2)

These statements of Islamic and Christian scholars, showing that omission from burial traditions was not contradicting religious beliefs, were then made known to lower-ranking religious leaders and the public.

#### Focus on One Health Topic

During the Ebola epidemic, public health discourses concentrated almost exclusively on the Ebola virus. Moreover, as the whole population was affected by the epidemic in similar ways, messages were standardized to ensure consistency and fight misinformation. As these messages were short and simple, they eased memorization of the messages for non-medical messengers:

We were confused and the messages were not consistent by then [initial months of the epidemic]. Then they designed a single message and this message was shared. If we are talking about testing today, it would be the same message tomorrow. If we are talking about ambulance service here, it is going to be the same message over there. (Opinion leader, M6)

In addition, all opinion leaders in this study mentioned trainings in which they received background information about the messages and were able to ask questions for clarification. Some were also supervised in their role as health messengers, which further helped them deliver consistent, reliable messages.

#### Available Funding

The Ebola outbreak was supported by considerable amounts of international and national funding. This allowed for repeated training for all levels of religious leaders and supervision of message consistency:

During Ebola, we got much funding, so we were able to do those types of trainings. Apart from the trainings, we also had meetings and we did field visits. …. We provided space for these religious leaders to come to the office to hold monthly meetings with our staff to get some orientation. (Developer, M2)

Moreover, the available funding also allowed for stipends for religious leaders, which further incentivized their willingness to spread health messages.

#### Integration into Well-Functioning Response Mechanisms

Once acknowledged as key opinion leaders with influence over their congregations, religious leaders became an integral part of the response. Due to their multitude in the country, they scaled up the number of available messengers and contributed to strengthening trust in other messengers and response mechanisms that had been unknown to the local population before the epidemic:

What really worked for Ebola is that people were hearing one set of things on the radio and then they were hearing the same thing in face-to-face conversations and they were hearing the same thing from their traditional leaders and religious leaders. (Developer, M7)

At the same time, religious leaders and the messages they disseminated also benefitted from being backed by those other response mechanisms, especially legal force, which ensured the compliance with required behavioral changes:

It wasn't taken up as a safeguard – it was a do-or-die-situation. You had no choice but to practice what the messages said. …. Whenever you're forced to do something, it also helps the message and the messenger in some way. (Developer, M8)

#### Need for Hope in Times of Despair

While religious leaders were not the only opinion leaders employed as health messengers, their role was emphasized by all participants for the hope they spread alongside Ebola risk messages. Religious leaders believed that packaging health messages in a sermon helped to calm people down and make them more likely to accept the messages:

They [local citizens] felt they are finished, there was no hope for them, but we gave them hope. We brought passages in the Bible wherein people lost hope but God restored them. And so we gave them precautions, sensitization, awareness-raising, and we gave them hope. Hope of eternity and hope in the present world. (Religious opinion leader, M1)

We asked ourselves ‘what kind of disease is this, why is God punishing us?’ But then our leaders came to pray with us. They told us to wash our hands and to trust in God. (Religious member, M27)

### RQ2: Non-emergency Setting

Due to their success in strengthening the Ebola response and increasing trust in health messages, religious leaders were subsequently integrated into the country’s long-term health promotion strategy (see Government of Sierra Leone, 2016). However, many contextual factors have changed, giving rise to several challenges concerning the role of religious leaders as health messengers. Due to *conflicting topics and priorities* (leadership), *a multitude of health topics with complex messages* (wider environment) and an *ambivalent reputation as health messengers* (organizational structures), the persuasiveness of health messages disseminated by religious leaders appears to have been weakened. These factors were further supported by an overall *lack of funding* (financial resources), *few legal regulations and inconsistent services* (policy), and the public’s *ambiguity of accepting fate versus taking action* (patients/public). Nonetheless, it should be noted that in the views of participants of all samples, the advantages of including religious leaders as health messengers outweighed the risks. Moreover, the incorporation of religious bodies in health communication, and thus, dialoguing with them was seen as an essential strategy to mitigate risks of unfavorable contextual features.

#### Ambivalent Reputation as Messengers for Health Information

Due to their role as health messengers during the Ebola epidemic, members of religious congregations were familiar with receiving health information from their religious leaders, a role that was previously not associated with religious teachings:

Yes, we already know him for giving us messages for staying healthy because he used to do this during the Ebola. We trust him, he has never lied to us. (Religious member, F34)

While religious leaders were largely described as trusted during the epidemic, ambivalences regarding their expertise in health matters and aptness to spread health information were mentioned for a non-emergency setting:

He is not a doctor and he has not studied it [medicine]. I don’t know about his knowledge. Shouldn’t each profession stay with their own? (Religious member, M50)

Their ambivalent reputation was further explained in relation to the following themes, conflicting topics, and a multitude of health topics.

#### Conflicting Topics and Priorities

Religious leaders in this study said they felt confident talking about any health topic, because of their ‘onus and responsibility to protect our congregations and to help the health workers’ (Religious opinion leader, M1). While the Islamic leaders remained vague concerning the health topics they would address, the two Christian leaders mentioned controversial and taboo topics they had addressed in the past (e.g., female genital mutilation). Because conflicting health topics (especially sex-related behaviors) are often deeply intertwined with religious and cultural values, the Christian leaders perceived themselves to be in a prime position to change those and be congruent with biomedical views. In contrast to these views, developers and non-religious opinion leaders noted that merging scientific with religious beliefs was not yet the norm and that, due to their influence, religious leaders should also be viewed as gatekeepers who could contribute to upholding the status quo:

If you talk on the issue of family planning, you talk about the use of condom. Like for the Catholic priests, they usually frown at it, you know, because according to the doctrine of the Catholic church, they don’t promote this kind of stuff. (Developer, M10)

Some opinion leaders noted that biomedical, scientific thinking was viewed as a Western practice and not viewed as necessarily compatible with religious views:

Some of those religious leaders have, especially like in the provincial areas, they have that perception that they are being indoctrinated and oppose these Western ideas, for example in the area of vaccination. (Religious opinion leader, M3)

Moreover, religious leaders in Sierra Leone traditionally earn their living from donations and the performance of spiritual healings, though the latter have lately been discouraged in favor of biomedical healthcare. A pastor explained that with his increasing understanding of biomedical views of health, he prayed for the person, but then made referrals to the healthcare system and refrained from performing paid spiritual healings. Since few incentives were given to religious leaders for disseminating health messages, refraining from healings was met with resistance from some of his colleagues:

Because some of my colleague pastors get their source of livelihood from this, they make things fearful to the people, so that they [religious congregations] will come to them. …. Some of them told me that the White man has indoctrinated me. (Religious opinion leader, M3)

While religious leaders in our sample expressed openness to merging their religious beliefs with scientific, biomedical views, to speak about taboo topics, and acknowledge the boundaries of their influence, the majority of other opinion leaders and developers had experienced opposition and problems in relation to health topics that conflicted with religious beliefs.

#### Multitude of Health Topics with Complex Messages

With the epidemic’s end, formerly neglected health topics became relevant again and increased the number of health messages to be disseminated. Amid a lack of biomedical training among religious leaders, understanding, memorizing, and conveying the correct health messages may be jeopardized, as several developers asserted:

To be honest with you, most of us know little about medicine and some even have very little general education and find it hard to read. It will be difficult for them to remember all the messages and bring them to their congregations. And for the people, too, because who can read and write and know if the message is right? (Opinion leader, 6)

Moreover, developers also pointed out differing needs among target audiences, which further increased the messages’ complexity:

People are different and not everyone experiences the same health problems. For other people again, you need to target them indirectly …. It’s quite difficult to have messages that appeal to different sets of people. (Developer, M7)

#### Lack of Funding

In a non-emergency setting, participants from all three samples noted a chronic lack of funding, the overall poor infrastructure in Sierra Leone, and the resulting difficulties disseminating health information regularly and equally to all members of society. Against these challenges, the dense network of religious institutions with community-based religious leaders was seen as a readily available resource for the dissemination of messages at low cost:

Because if one is not a Muslim, then he is a Christian and because of that whenever a message is being passed from the religious leaders, the people receive it. (Religious member, M37)

However, in contrast to the many trainings during the Ebola epidemic, workshops and trainings have also become scarce amid the lack of funding. Various developers described it as risky that, after short training sessions or the reception of a manual, religious leaders were sent off with no supervision to disseminate health messages. As religious leaders have no professional medical training, distortions and incomplete messages had been observed by various developers and other opinion leaders. A medical worker described how this had an impact on how other messages were perceived:

So, because you have got that wrong message from those religious leaders, when we the health workers are going to give them messages, they will say ‘No, we have been practicing this one.’ We have been getting challenges in terms of them accepting the view of the health workers. (Opinion leader, M7)

#### Few Legal Regulations and Inconsistent Services

Apart from a few laws that regulate certain health behaviors (e.g., a legal requirement to deliver babies in a healthcare facility and not at home), most health behaviors in a non-emergency setting are recommendations and rely on voluntary behavior change. This way, the positive outcomes of behavior change are less observable and convincing:

You need to have a level of education and patience to see, if I do this, this and this has improved. But tell me, who knows at community level that Y leads to Z? …. So, telling people what they can’t observe can also affect how they see the messenger. (Developer, M9)

Moreover, while the health services promoted were largely functioning during the Ebola outbreak, services since have become less effective, which affects the credibility of the messenger:

They [religious leaders] told us ‘If you know services are not going, if you know services are not at this, don’t tell us to go and say it because we have our congregation, we don’t want to lose their trust.’ (Developer, M1)

#### Ambiguity of Accepting Fate Versus Taking Action

Religious leaders and various developers in this study pointed out the interconnectedness of religious beliefs and taking care of one’s health and, thus, their responsibility to speak about health and encourage health-protective behaviors:

If you are a believer in Allah, you will do the things that will protect yourself according to the directives you receive from the teachings from Islam. One should not say ‘I leave everything to God.’ (Religious opinion leader, M2)

I warn them, ‘Don't put the Lord, thy God to test!’ If they say, ‘I will not go to the hospital, there is God.’ God will not come from heaven and solve the problem for you – you take the necessary precautions to avert the trouble. (Religious opinion leader, M1)

However, there was also agreement that there is an inherent tension among believers of any faith between accepting one’s fate as predetermined by the will of God and not accepting it as unchangeable and, thus, taking action to influence it. Especially among the many poor in Sierra Leone who can exercise little control over the risks they experience daily (e.g., being too remote from a healthcare center to seek care in an emergency or having a child with a disability), daily prayers for good health or health improvements were recurringly described as the only option in all focus groups:

We just pray and by God in power, God will protect us and give us long life. (Religious member, F11)

God can send sicknesses as punishment, like this Ebola, it was a plague from God. So you have to pray. (Religious member, M15)

Submitting one’s health to God was seen as a way of coping as well as explaining health conditions.

Nonetheless, in other matters, those same leaders also expected their religious communities to ‘accept that I need to bear with my own [fate] and exercise patience’ (Religious opinion leader, M3). In the views of the religious leaders, it thus depends on circumstances whether they recommend submitting to God or encouraging action. However, it also became evident that a leader’s exposure to biomedical knowledge had an influence on how they perceived and framed the locus of control of the person affected:

We should have a broader perspective because there are diseases which we [religious leaders] don’t know about, but the scientists do. We need to have that medical idea, so we can tell the person ‘Please, go to the hospital.’ (Religious opinion leader, M3)

Hence, while members of religious communities expressed a willingness to improve their health, they often felt powerless to influence it. Accepting their fate and asking their religious leaders for prayers were oft-described methods of dealing with sicknesses. In the case of preventable or treatable conditions, religious leaders were ascribed a key responsibility as they could, on the one hand, encourage healthcare-seeking or preventive action or, on the other hand, strengthen fatalistic perceptions and discourage further action.

## Discussion

To explore the contextual factors of emergency and non-emergency settings in relation to the inclusion of religious opinion leaders as health messengers, this study used a qualitative approach and triangulated the perspectives of three different samples, including (religious) opinion leaders, members of religious communities, and developers of health communication strategies. Aligning our findings with previously described context factors of health-campaign planning frameworks (Nilsen & Bernhardsson, 2019), we provide multifaceted contrasts between context factors during a public health emergency versus a non-emergency setting and their meaning for the inclusion of religious leaders as messengers.

### Comparison of Contextual Features During Emergency and Non-emergency Setting

Our data indicate that the contextual features of a public health emergency can be greatly beneficial in embedding religious leaders in health campaigns among populations with high religious affiliations, a finding that has also been confirmed during the COVID pandemic (Isiko, 2020; Viskupic & Wiltse, 2021). As religious leaders are locally known beyond their respective congregations, they can be identified quickly to target the local population in their proximity. Using standardized messages spread by multiple messengers, religious leaders confirm the credibility of other messengers and likewise benefit from being reconfirmed by others, which can enhance their position as a reliable source of health information. Moreover, by spreading hope and encouragement alongside health messages, religious leaders appear to be in a unique position to make audiences susceptible to process and comply with required behaviors. The backing of legal force and a concentration on one health topic further strengthened this strategy during the Ebola outbreak.

In contrast, in a non-emergency setting, most health behaviors are less legally regulated, and religious leaders receive little training and deal with multiple health topics. These factors give rise to a range of potential risks when employing religious leaders as health messengers, e.g., they can censor messages that may conflict with religious beliefs or their need to make a living from spiritual healings. Nonetheless, continued integration of religious leaders as health messengers was also viewed as a strategy to expose these leaders to biomedical views, increase their health literacy, and thus mitigate some of the risks.

### Theoretical and Practical Implications

With regard to theoretical implications, our study resonates with the work of other scholars. On the one hand, we could show the positive implications of being religious for mental well-being especially in times of stress and hopelessness (You et al., 2019). On the other hand, we have also shown how religious leaders may prevent members of their communities from adopting health-protective behaviors if these do not align with religious doctrines or the leader’s personal agenda (Costa et al., 2020). Moreover, our study provides a unique and nuanced spectrum of the ambivalences of religious leaders in health communication in emergency and non-emergency settings in a population with high religious affiliations. The multifaceted theoretical implications are also reflected in the comprehensive methodological approach that combined multiple perspectives on the topic of interest.

In addition, our findings have several implications for practice and policy. First, the inclusion of religious leaders in the response to public health threats should be prioritized due to their impact on mental well-being in times of stress and required immediate behavior change. Second, the willingness of religious leaders to address controversial topics and to refrain from spiritual healings in favor of medical treatment appears to be influenced by the leader’s exposure to schools of thoughts outside the religious realm. High-level religious leaders such as a sheikh or bishop who were said to be more educated and experienced in merging their religious with scientific views can potentially be influential opinion leaders to lower-ranking, community-based religious leaders. This is not to say that spiritual healings are incompatible with biomedical views; instead, the merging of both perspectives can serve as reflections of the boundaries of both healthcare and spiritual healings, as well as how they enhance each other for mental and physical well-being. Written-down health messages supported by religious scriptures can further support the compatibility of religious and biomedical perspectives and convince religious leaders and their audiences alike. Third, as religious leaders have no medical background, providing comprehensive and at the same time short, memorable health messages on multiple topics remains a challenge and bears the risk of inconsistency. Proper training and supervision appear vital to ensuring that messages are understood and delivered as intended. Various participants referred to messenger services such as WhatsApp as a low-cost technology to enable sharing more complex messages via audio and to supervise remote community-based religious leaders. This way, supervisors can also become aware of message rejections or other issues with topics and address them with the leader before potential negative effects for religious communities are felt. Last, despite the advantages of relying on the dense network of religious leaders as health messengers, religious leaders should not be an exclusive source of information. In both emergency and non-emergency settings, the reputation of religious leaders benefits from being backed by other information sources, especially from the medical sector, and vice versa.

### Study Limitations

This study is not without limitations. Research was carried out in Sierra Leone and our findings might not be entirely transferable to a different setting. For instance, while religious leaders were also integrated into Ebola response mechanisms in Liberia and Guinea (Marshall, 2017), we are not aware of any policy changes that included religious opinion leaders in public health measures beyond the epidemic in these countries. Sierra Leone might also be exceptional in terms of how the country’s high religious tolerance and openness to merge religion with other belief systems (Day, 2021) creates favorable circumstances for religious leaders as health messengers. Moreover, the proposed practical implications and their effects require testing in future studies, preferably with a longitudinal design. There are also methodological limitations. Although we aimed to recruit participants with distinct perspectives, our study has likely been affected by selection bias, especially among the religious leaders, as all of them were highly supportive of blending religious teachings with health messages. A future study with only religious leaders in the sample of interviewees might allow further insights into variances between religious leaders. Despite thorough recruitment efforts to include a balanced sample of male and female interview participants, most interviewees were male. This has likely affected the views presented in our results. Last, the first author’s cultural background, distinct from those of the majority of participants, may have had an impact on how participants expressed their views as well as on the analysis of the data.

## Conclusion

Although the influential role of religious leaders in health communication has received considerable attention, a nuanced analysis of the contextual factors and resulting risks and opportunities of the inclusion of these leaders has been neglected in the literature. By proposing several recommendations for the incorporation of religious leaders in health promotion activities, our study supports the promising potential of religious leaders as health messengers.

## Data Availability

The datasets used and/or analyzed during the current study are available from the corresponding author on reasonable request.
